# Free and Bioavailable Fractions of Vitamin D: Association with Maternal Characteristics in Brazilian Pregnant Women

**DOI:** 10.1155/2020/1408659

**Published:** 2020-09-16

**Authors:** Joana N. Pereira, Julia Chactoura, Fernanda Nohra, Maria Eduarda L. Diogenes, Flávia F. Bezerra

**Affiliations:** ^1^Instituto de Nutrição, Universidade do Estado do Rio de Janeiro, Rio de Janeiro, Brazil; ^2^Instituto Nacional de Câncer José Alencar Gomes da Silva, Rio de Janeiro, Brazil

## Abstract

Normal pregnancy is characterized by many changes in vitamin D metabolism, challenging the assessment of vitamin D status based exclusively on serum total 25-hydroxyvitamin D (25(OH)D). We hypothesized that measuring free and bioavailable fractions contributes to a better vitamin D status assessment in late pregnancy. Our aim was to evaluate a broad set of biomarkers of vitamin D status in Brazilian women in the third trimester of pregnancy. This cross-sectional study was conducted in women (*n* = 123, 18–44 y, 27–41 wk gestation) attended in a public maternity in Rio de Janeiro (2016–2018). Biomarkers included serum concentrations of total 25(OH)D_3_, parathyroid hormone (PTH), vitamin D-binding protein (DBP), and free and bioavailable fractions of 25(OH)D_3_. Vitamin D insufficiency (<50 nmol/L) was prevalent in 47.9% of the pregnant women. Serum 25(OH)D_3_ was inversely associated with the gestational week (*β* = −0.71, 95% confidence interval (CI): −1.31 to −0.16) and season, being lower in autumn (*β* = −9.90, 95% CI: −16.14 to -3.64) and winter (*β* = −16.74, 95%CI: −23.13 to −10.34). Concentrations of DBP, and free and bioavailable 25(OH)D_3_ were also inversely associated with winter months (*P* < 0.05). DBP was directly associated with prepregnancy BMI (*β* = 5.84, 95% CI: 0.62 to 11.06). The recognized season-effect on total 25(OH)D_3_ appeared to also occur on free and bioavailable fractions. Although advanced gestational age was associated with lower total 25(OH)D_3_, our results suggest an adaptive mechanism responsible for maintaining free fraction during the 3^rd^ trimester. We also suggest that starting pregnancy in obese condition may have an impact on vitamin D bioavailability, which deserves further investigation.

## 1. Introduction

It is estimated that inadequate vitamin D status affects about 1 billion people worldwide [1, 2]. Pregnant women have been considered one of the main risk groups for vitamin D insufficiency due to increased demand to ensure fetal bone mineralization. Moreover, observational studies suggest an increased risk of gestational diabetes, preeclampsia, and small for gestational age infants in vitamin D insufficient mothers [3–5].

The assessment of nutritional status of vitamin D is usually based on serum measurements of its main circulating metabolite, 25-hydroxyvitamin D (25(OH)D). About 85–90% of the total serum 25(OH)D circulates bounded to vitamin D-binding protein (DBP), 10–15% are loosely bound to serum albumin, and the minority fraction of 25(OH)D is unbound in free condition [6, 7]. The terminology “bioavailable 25(OH)D” has been used to represent the sum of the albumin-bound and free fractions. Although subjected to an intense debate in the last years, a serum total 25(OH)D cutoff value of 50 nmol/L for defining insufficiency in general population has been relatively well accepted by scientific community [8]. More recently, based on the “free hormone hypothesis” [9], it has been proposed that measuring both free and bioavailable fractions may be of clinical relevance, as it is for other hormones (e.g., thyroid and sex hormones) and nutrients (e.g., retinol) [9, 10]. This may be of particular importance when evaluating vitamin D status in situations in which vitamin D homeostasis is subjected to physiologic adaptations, as observed during pregnancy.

Serum concentrations of total 25(OH)D appear to remain unchanged or slightly decreased during pregnancy [4, 10, 11]. A progressive increase in serum concentrations of the biologically active metabolite 1,25-dihydroxyvitamin D (1,25(OH)_2_D) is well documented [10, 12] and is considered the main physiologic adaptation to promote an increase in calcium absorption, especially during the third trimester [13], and to ensure fetal bone mineralization [14, 15].

There is also evidence of an increase in concentrations of DBP during pregnancy that is attributed to a rise in liver synthetic function in response to the high estrogen concentrations in this period [7, 16, 17]. The reported magnitude of this increase is, however, quite variable [6, 7, 10]. Furthermore, decreases in concentrations of free and/or bioavailable fractions of 25(OH)D during pregnancy were observed in some [9, 16] but not all [10] studies. Different methods used for measurement of DBP (mono- *vs*. polyclonal antibody immunoassay) and vitamin D fractions (direct measurement *vs*. estimation by equation) can contribute to partially explain the high variability reported [9, 10, 16]. Nevertheless, differences in population characteristics may also matter.

The few studies estimating free and/or bioavailable 25(OH)D during pregnancy were conducted mainly in Caucasians, with none of them conducted in mixed population such as Brazilians. We hypothesized that measuring free and bioavailable vitamin D fractions contributes to a better vitamin D status assessment in late pregnancy. The aim of this study was to evaluate a broad set of biomarkers of vitamin D status in Brazilian women in the third trimester of pregnancy, as well as their association with maternal characteristics.

## 2. Methods

### 2.1. Study Design and Population

This cross-sectional study was conducted at a public maternity in Rio de Janeiro (22°57′S) between October 2016 and March 2018. The study started after written informed consent of the participants previously clarified about the purpose and procedures adopted in the study. The study protocol was registered at the Brazilian National Research Ethics System and approved by the Ethical Committee of the Rio de Janeiro State Secretariat for Health. Exclusion criteria were as follows: pregnant women under 18 years old, multiple gestation, high-risk pregnancy (hypertensive disorders, including preeclampsia diagnosis, and gestational diabetes), diagnosis of chronic disease, and disturbance in renal and liver functions.

After exclusions, a total of 123 Brazilian women, recruited during prenatal care at the third trimester of pregnancy (27–41 weeks), agreed to participate in this study. Participants were invited to perform an interview that included (1) a structured questionnaire to record sociodemographic and gestational information, (2) skin color assessment, and (3) blood sample collection. Information on maternal age, education level, gestational week, number of previous pregnancies, smoking habits, alcohol consumption, use of supplements, and exercise practice during pregnancy were collected during the interview. Information on season (spring, summer, autumn, and winter) was also registered at this time. Two participants had insufficient material for biochemical analyses, and two participants had unexplained very high (>2000 mg/L) DBP or PTH (>120 pg/mL) concentrations; thus, 119 participants with overall complete information were included in the current analyses.

The gestational period was categorized into the first (27–31 wk), middle (32–36 wk), and the last five weeks (37–41 wk, corresponding to the “term” period) of the third trimester. Information on the prepregnancy body mass index (BMI, in kg/m^2^) was collected from medical records at the first prenatal visit. Based on BMI before pregnancy, women were categorized in underweight, within normal range, overweight, or obese according to WHO criteria [18]. Gestational weight gain at the current prenatal visit was calculated based on self-reported prepregnancy weight and measured current weight. Information on the estimated weekly weight gain was used to divide the women in two categories according to the median value (below *vs*. equal to or above).

### 2.2. Blood Collection and Biochemical Analyses

Fasting blood samples (10 ml) were collected, and serum was separated by centrifugation and stored at −80°C until laboratory analysis. Serum concentration of total 25(OH)D_3_ was measured by high-performance liquid chromatography coupled with a diode array detector (HPLC-DAD), as previously described [19, 20], with slight modification. Briefly, serum was deproteinized with methanol: isopropanol (80 : 20 v/v) and 25(OH)D extracted with n-hexane. The n-hexane phase was collected, evaporated to dryness, and the residue dissolved in the mobile phase (methanol : water, 90 : 10, v/v). An Agilent HPLC 1260 Infinity system at a flow rate 1.0 mL/min, 25°C, and Kromasil® C-18, 4.6 × 300 mm, 5 *μ*m column was used. 25(OH)D_3_ was measured using a diode array detector (DAD) at 265 nm. The intra-assay coefficient of variation was <2%. Vitamin D status was defined based on 25(OH)D_3_ concentrations according to the Institute of Medicine's (8) cutoffs for adults: 25(OH)D_3_ < 50 nmol/L (insufficiency) and 25(OH)D_3_ ≥ 50 nmol/L (sufficiency). Intact parathyroid hormone (iPTH) was measured by the enzyme-linked immunoabsorbent assay (ELISA; ALPCO Diagnostics, Salem, NH). The inter- and intra-assay coefficients of variation were <7% and <6%, respectively. Serum albumin was measured using an enzymatic colorimetric method (Bioclin, Brazil). The inter- and intra-assay of variations were below 3%. DBP concentration was measured using a polyclonal antibody commercial ELISA kit (Immundiagnostik AG, Bensheim, Germany). The inter- and intra-assay coefficients were <7% and <5%, respectively. Additionally, in order to expand comparability, DBP was also measured in a subsample (*n* = 52) by an alternative monoclonal antibody ELISA kit (R&D Systems; Bio-Techne, Minneapolis, USA).

Free and bioavailable fractions of 25(OH)D_3_ were indirectly estimated based on the protein-ligand binding kinetics. Free 25(OH)D_3_ was calculated using 25(OH)D_3_, DBP, and albumin concentrations and the binding affinity constants of 25(OH)D_3_-DBP (7 × 10^8^ M^−1^) and 25(OH)D_3_-albumin (6 × 10^−5^ M^−1^) [21, 22]. Bioavailable 25(OH)D_3_ (referred as bio25(OH)D_3_) corresponds to the non-DBP bound vitamin D fraction and was calculated using the information of free 25(OH)D_3_ as well as concentration and binding affinity constant of albumin [22].

### 2.3. Skin Color Measurements

Constitutive skin pigmentation was measured using a portable reflectometer (Chroma Meter CR-400, New Jersey, EUA) at the upper right armpit, a site usually not exposed to sunlight, and used to classify skin type into six groups according to individual typology angle (ITA): very light, light, intermediate, tanned, brown, and dark [23, 24]. Few pregnant women were classified as “very light” skin type (*n* = 2) and, therefore, were grouped in the category of “light” skin type. The pregnant woman (*n* = 1) classified with “very dark” skin was grouped in the category of “brown” skin, resulting in four constitutive skin type categories (very light or light, intermediate, tanned, and brown or dark). Facultative pigmentation was determined from measurement on the dorsum of the right hand, a site generally exposed to sunlight. The values of luminance (^*∗*^), which ranges from 0 (absolute black) to 100 (absolute white), were recorded and used to estimate the sun exposure index (SEI). The SEI is used as an indicator of cumulative UV exposure and was calculated from the difference between facultative and constitutive pigmentation [25].

### 2.4. Statistical Analyses

Data were presented as mean ± standard deviation (SD) for continuous variables and frequencies and percentages (%) for categorical variables. ANOVA followed by the least square means (LSD) post hoc test was used to compare 25(OH)D_3_, DBP, PTH, and free and bioavailable 25(OH)D_3_ means by categories of independent variables. Pearson's correlation analysis was conducted to investigate associations between all biochemical parameters. Based on information reported in the literature [26, 27], the following variables were investigated as potential factors associated with 25(OH)D_3_, DBP, and free and bioavailable 25(OH)D_3_: maternal age, gestational week, prepregnancy BMI, gestational weight gain, number of previous pregnancies, skin color (constitutive, ITA value, and facultative, SEI value), and season of blood sample collection (for south hemisphere: winter, June-September; spring, September-December; summer, December-March; autumn, March-June). All these variables were mutually adjusted in a multiple linear regression analysis to investigate the determinants of 25(OH)D_3_, DBP, PTH, and free and bioavailable 25(OH)D_3_. With the exception of season, that was a dummy variable, all the others were included in the regression as continuous variables. In a subsample (*n* = 52), a paired *t*-test was conducted to evaluate differences in serum DBP concentrations using the polyclonal antibody and the alternative monoclonal antibody ELISA assay. Statistical analyses were performed using the SPSS 22.0 software (version 22, Inc., Chicago, IL, USA), and statistical significance was set at *P* < 0.05.

## 3. Results

Descriptive characteristics of the study population are shown in Table 1. Pregnant women were between 18 and 44 y old and were all at the 3^rd^ trimester of gestation (27–41 wk). Based on prepregnancy BMI, more than half (∼55%) of the participants were within the normal weight range before pregnancy [18]. The majority of women had one or more previous gestations (69%), had at least complete fundamental education level (88%), reported no cigarette smoking (93%), no alcohol consumption (88%), nor physical exercise (92%) during current pregnancy. Only one woman reported use of multivitamin supplement containing vitamin D. Blood sampling was carried out throughout the year, in order to balance sample distribution between the four seasons. They were also well distributed in the four skin type categories classified according to ITA values (Table 1).

Total serum 25(OH)D_3_ concentration ranged from 24.9 to 85.7 nmol/L (mean ± SD: 50.7 ± 13.9 nmol/L), and vitamin D insufficiency (<50 nmol/L, 8) was found in 47.9% of the pregnant women. DBP concentrations ranged from 491.2 to 1302.8 mg/L (mean ± SD: 778.0 ± 148.1).

Serum concentrations of free and bioavailable 25(OH)D_3_ fractions ranged from 0.58 to 2.66 pg/mL and from 0.14 to 0.74 ng/mL, respectively. These values represented about 0.007% and 1.9% of the total 25(OH)D_3_ concentrations, respectively. Mean PTH and albumin concentrations were 29.5 ± 14.9 pg/mL and 3.12 ± 0.42 g/dL, respectively.

Unadjusted means of 25(OH)D_3_, DBP, PTH, and free and bioavailable fractions of 25(OH)D_3_ were described by categories of gestational week, prepregnancy BMI, skin type, number of previous pregnancies, gestational weight gain, and season of the year (Table 2). Serum DBP concentrations were ∼9% higher in the beginning of the third trimester compared to the subsequent weeks, although mean differences did not reach statistical significance (*P*=0.062). DBP was also ∼10% higher in women that started pregnancy in obese condition compared to overweight and normal women, again with no statistical significance (*P*=0.072). Based on the prepregnancy BMI, obese women had lower mean free 25(OH)D_3_ concentrations compared to those who were overweight before pregnancy (1.20 ± 0.31 and 1.48 ± 0.42, respectively; *P*=0.038 for pairwise comparisons) (Table 2).

Serum total 25(OH)D_3_, DBP, and free and bioavailable fractions of 25(OH)D_3_ concentrations were influenced by season of the year (Table 2). Compared to women who had blood collected during spring months, those who were studied during autumn and winter months had 17% and 30% lower 25(OH)D_3_ concentrations (*P*=0.008 and *P* < 0.001, respectively). Winter was also associated with lower concentrations of DBP and free and bioavailable fractions of 25(OH)D_3_ (*P* < 0.05). There were no statistically significant differences in all biochemical markers among categories of gestational week, skin type, number of previous pregnancies, and gestational weight gain.

Correlations between biochemical measurements were explored by Pearson's correlation analysis. Serum total 25(OH)D_3_ concentrations were directly associated with albumin (*r* = 0.320, *P* < 0.001) and DBP concentrations (*r* = 0.231, *P*=0.01). Albumin was also directly associated with DBP (*r* = 0.267, *P*=0.004) and with bioavailable 25(OH)D_3_ concentrations (*r* = 0.446, *P* < 0.001). As expected, free and bioavailable 25(OH)D_3_ fractions were directly associated with total 25(OH)D_3_ (*r* = 0.719 and *r* = 0.764, respectively, both *P* < 0.001) and inversely with DBP (*r* = −0.439, *P* < 0.001 and *r* = −0.287, *P*=0.002, respectively). No statistically significant association was observed between 25(OH)D_3_ and PTH.

Multiple linear regression analyses were performed to investigate the potential exposure variables (each one adjusted by all the others) associated with total 25(OH)D_3_, DBP, PTH, and free and bioavailable 25(OH)D_3_ concentrations (Table 3). Gestational week was inversely associated with serum total 25(OH)D_3_, with an estimated decrease of 0.73 nmol/L with each additional week of gestation. The prepregnancy BMI was directly associated with DBP concentrations (*β* = 5.92, 95% CI: 0.74, 11.09). Taking spring as the reference season, collecting blood in autumn months was associated with lower serum concentrations of both serum total 25(OH)D_3_ (*β* = −9.89, 95% CI: −16.14, −3.64) and serum DBP (*β* = −86.78, 95% CI: −158.77, −14.79). Collecting blood in winter months was associated with lower serum concentrations of serum total 25(OH)D_3_ (*β* = −16.74, 95% CI: −23.13, −10.34), DBP (*β* = −85.39, 95% CI: −159.10, −11.69), free 25(OH)D_3_ (*β* = −0.33, 95% CI: −0.53, −0.13), and bioavailable fraction of 25(OH)D_3_ (*β* = −0.11, 95% CI: −0.17, −0.05). A higher SEI, a marker of skin tanning, was inversely associated with serum PTH levels (Table 3). After adjustment for all the other exposure variables, gestational weight gain, age, ITA, sun exposure index, and the number of previous pregnancies were not associated with any biochemical measurements.

In a subsample, it was observed that DBP measured by the monoclonal antibody (*m*-DBP) was significantly lower than DBP measured by the polyclonal (*p*-DBP) antibody assay (347.3 ± 63.3 mg/mL vs. 773.5 ± 129.5 mg/mL, *P* < 0.001). Mean free and bioavailable 25(OH)D_3_ estimated using results from *m*-DBP in the equation were both 55% higher than the estimated concentrations obtained from *p*-DBP (supplemental material).

## 4. Discussion

The assertive determination of vitamin D status in the setting of pregnancy is of particular importance due to the potential simultaneous implications for both maternal and fetal health [3, 28, 29]. Given that normal pregnancy is characterized by many changes in vitamin D metabolism, the determination of vitamin D status based exclusively on the 25(OH)D concentrations has been questioned [9]. In the present study, serum concentrations of DBP, and free and bioavailable fractions of 25(OH)D_3_ were described for the first time in Brazilian women at the last pregnancy trimester. Our results suggest that the recognized season-effect on total 25(OH)D_3_ appears to also occur on free and bioavailable fractions. Although the progression of pregnancy at the last trimester was associated with lower total 25(OH)D_3_, our findings suggest that maternal organism is able to maintain free 25(OH)D_3_ fraction independent on gestational age.

The impact of pregnancy on the concentrations and bioavailability of 25(OH)D is not completely understood; nevertheless, the criteria for defining vitamin D status in pregnant women are the same used for general adult population. In the present study, vitamin D insufficiency reached 47.9% of women. Adopting the cutoff value of 50 nmol/L for total 25(OH)D [8], a wide global variation of vitamin D insufficiency has been observed in pregnant women, varying from 46% in Eastern Europe to 87% in East Asia [30]. A number of environmental and individual intrinsic factors may contribute to the great variability in the prevalence of vitamin D insufficiency, including season and latitude of residence, as well as differences in the genetic background of the populations, skin pigmentation, and adiposity status [26, 31, 32]. In Brazil, the few studies conducted during pregnancy also reveal a great variation in the prevalence of vitamin D insufficiency. A study with adolescent women during the 3^rd^ trimester of gestation showed 43% of the mothers with vitamin D insufficiency [33]. A more recent prospective cohort conducted in the same municipality and same gestational period revealed that vitamin D insufficiency was prevalent in only 10.2% of the pregnant adult women studied [34].

The determinants of vitamin D deficiency in the third trimester of pregnancy (36–42 wk) were recently investigated in a multicentre study in Switzerland [27]. The authors observed that the centre of study, country of origin, season of delivery, and vitamin D supplement intake were the main determinants of vitamin D status, while BMI, skin color, use of sunscreen, and education level were not individually correlated with vitamin D concentrations. Furthermore, some data suggest that the 25(OH)D concentrations may be modulated by pregnancy-related factors, including the gestational age. In a longitudinal study conducted in 1768 pregnant women (15 to 40 wk gestation), the progression of gestation was inversely associated with 25(OH)D concentration, with a nadir at 36 weeks [4].

In the present study, conducted in the 3^rd^ trimester of gestation (27–41 wk), the main factors associated with total 25(OH)D_3_ concentrations were season of blood sample collection and gestational week, with lower concentrations being observed among women participating in the study during autumn/winter months and in those in the more advanced weeks of gestation. Of note, season was a determinant factor though pregnant women reside in a region with tropical climate and, therefore, without well-defined seasons throughout the year.

Although season-effect on vitamin D status in the general population is well known, its influence on serum concentrations of DBP as well as on 25(OH)D free and bioavailable fractions is still not clear [15, 26, 35]. In a case-control study with African and European American women, DBP had an opposite seasonal trend to total 25(OH)D, with concentrations being higher during spring and winter and lower in during summer and autumn [26]. In a longitudinal study with healthy blood donors, while there were no seasonal variations in serum DBP concentrations, free and bioavailable 25(OH)D concentrations followed the same seasonal variation of total 25(OH)D, with a peak during the summer and a low point during the winter [35]. Similar results were reported in a longitudinal study conducted in pregnant women that observed seasonal variations during 2^nd^ and 3^rd^ trimesters in serum total 25(OH)D and in its free and bioavailable fractions, but not in DBP concentrations [15]. In the present study, not only total 25(OH)D_3_ but also DBP and fractions of 25(OH)D_3_ concentrations showed the same seasonal-dependent pattern, with higher concentrations being observed in women whose blood sample was obtained during spring and lower in those during winter.

It is noteworthy that free 25(OH)D_3_ values were all within the reference interval proposed by Tsuprykov and co-workers [7] for calculated free 25(OH)D. Also, the relative proportions of free and bioavailable 25(OH)D_3_ fractions (defined as a percentage from total 25(OH)D_3_), were not different among the three categories of gestational week studied. Our findings suggest that maternal organism is able to maintain the percentage of free 25(OH)D_3_ fraction/total 25(OH)D_3_ independent on gestational age. DBP concentrations were directly associated with 25(OH)D_3_ suggesting a control mechanism for maintenance of the relative free and bioavailable 25(OH)D_3_ concentrations even under conditions of lower circulating 25(OH)D_3_. Consistently, there were no differences in absolute neither relative (%) concentrations of free or bioavailable 25(OH)D_3_ between vitamin D sufficient *vs*. insufficient pregnant women.

DBP concentrations in the present study were also directly associated with prepregnancy BMI. Although studies have reported that obesity disfavors an adequate 25(OH)D concentration [36–38], the association between obesity and other biomarkers of the vitamin D status was poorly investigated. Higher DBP concentrations in obese individuals have been previously reported [39, 40]. It was postulated that the higher interleukin-6 frequently observed in obesity could be behind the higher DBP concentrations among obese individuals [39]. Alternatively, it is also speculated that higher levels of free estrogen in obese people could stimulate hepatic synthesis of DBP [40]. High DBP concentrations during pregnancy have been observed and are consistent with the hyperestrogenic state typical of pregnancy [7]. We hypothesized that DBP levels could be even higher in pregnant women that started pregnancy with obesity, which in turn may result in lower bioavailability of vitamin D. Nevertheless, the only longitudinal study conducted in pregnant women addressing this question found no significant association between BMI during pregnancy and serum concentrations of DBP, and total and free 25(OH)D_3_ [4].

It is important to consider that the data on the bioavailability of vitamin D is subjected to a great variability, especially when it is indirectly estimated based on the protein-ligand binding kinetics. In part, this variability can be explained by the use of different analytical methods for measuring the DBP concentrations, in particular ELISA techniques employing monoclonal (*m*-DBP) or polyclonal antibodies (*p*-DBP) [41]. In contrast to *p*-DBP, the *m*-DBP may not be reliable to measure the *GC*1F, the genetic DBP variant most frequently observed in black individuals [9, 42]. Considering the mixed racial characteristics of the Brazilian population and in order to expand comparability with the literature, we also analyzed *m*-DBP in a subsample (*n* = 52) of the pregnant women studied. As expected, *m*-DBP concentrations were significantly lower compared to *p*-DBP. As a consequence, the 25(OH)D fractions estimated by *m*-DBP were overestimated and did not appear to reflect the actual concentrations in Brazilian pregnant women.

The present study was limited by the sample size (*n* = 119) that was not designed to be representative of the pregnant Brazilian population and may have underpowered the detection of small effects. Also, serum 25(OH)D_3_ was analyzed by HPLC-DAD, although liquid chromatography-tandem mass spectrometry (LC-MS/MS) is considered the gold standard for measurement of 25(OH)D concentrations [43]. Additionally, serum concentrations of 25(OH)D_3_ fractions were estimated indirectly from *p*-DBP ELISA and, therefore, were subjected to accurate measurements of DBP and albumin [44, 45]. Other potential determinants of vitamin D status, such as diet intake or genetic background, were not investigated.

## 5. Conclusions

This study evaluated a broad set of biomarkers of vitamin D status in Brazilian pregnant women contributing with information on free and bioavailable fractions of vitamin D in this particular physiologic state. Although Brazil is a sunny country throughout the year, we found that seasonality is the main determinant not only of serum total 25(OH)D_3,_ but also of serum concentrations of DBP, and free and bioavailable 25(OH)D_3_ fractions. Although advanced gestational age was associated with lower total 25(OH)D_3,_ our results suggest the existence of an adaptive mechanism responsible for maintaining its free fraction during the third trimester of pregnancy. Higher BMI before pregnancy was an additional factor contributing to higher DBP concentration. The impact of pregnancy on the bioavailability of vitamin D, as well as its consequences for mother and fetal health, deserves further investigation, particularly for those who may be at greater risk of developing inadequate status of vitamin D, including obese pregnant women.

## Figures and Tables

**Table 1:** General characteristics of the pregnant women studied.

Characteristics	Participants (*n* = 119)^*∗*^
Age, y	26.3 ± 5.7
Gestational period, wk	33.9 ± 4.1
Categories of gestational period	
27–31 wk	37 (31.1)
32–36 wk	42 (35.3)
37–41 wk	40 (33.6)
Prepregnancy BMI	
Underweight or normal (<25 kg/m^2^)	67 (56.3)
Overweight (25–29.9 kg/m^2^)	25 (21)
Obesity (≥30.0 kg/m^2^)	27 (22.7)
Gestational weight gain, kg/wk	0.32 ± 0.15
Number of previous pregnancies	
0	37 (31.1)
1	48 (40.3)
≥2	34 (28.6)
Skin type^†^	
Very light or light	22 (18.5)
Intermediate	34 (28.6)
Tanned	32 (26.9)
Brown or dark	31 (26.1)
Cigarette smoking	
Yes	8 (6.7)
No	111 (93.3)
Alcohol consumption	
Yes	14 (11.8)
No	105 (88.2)
Physical exercise	
Yes	10 (8.4)
No	109 (91.6)
Education level	
Incomplete fundamental	14 (11.8)
Complete fundamental	53 (44.5)
Complete high school or more	52 (43.7)
Season	
Winter	28 (23.5)
Spring	36 (30.3)
Summer	27 (22.7)
Autumn	28 (23.5)
Vitamin D status	
Insufficiency (<50 nmol/L)	57 (47.9)
Sufficiency (≥50 nmol/L)	62 (52.1)

^*∗*^Values are mean ± standard deviation or number of observations (% frequency). ^†^According to the individual typology angle (ITA).

**Table 2:** Serum concentrations of vitamin D biomarkers according to general characteristics of the adult Brazilian pregnant women studied.^*∗*^

	25(OH)D_3_		DBP		PTH		Free 25(OH)D_3_		Bioavailable 25(OH)D_3_	
	(nmoL/L)	*P* ^†^	(mg/L)	*P*	(pg/mL)	*P*	(pg/mL)	*P*	(ng/mL)	*P*
Gestational period										
27–31 wk	54.4 ± 14.3	0.145	825 ± 164	0.062	28.7 ± 13.6	0.560	1.38 ± 0.40	0.679	0.40 ± 0.14	0.369
32–36 wk	49.4 ± 13.1		753 ± 146		31.5 ± 17.4		1.35 ± 0.38		0.37 ± 0.11	
37–41 wk	48.6 ± 14.1		760 ± 128		28.2 ± 13.3		1.30 ± 0.44		0.36 ± 0.13	
BMI before pregnancy										
Underweight or normal	49.5 ± 13.2	0.513	768 ± 153	0.072	28.4 ± 13.7	0.632	1.35 ± 0.42^ab^	**0.048**	0.38 ± 0.13	0.065
Overweight	53.3 ± 13.5		746 ± 144		30.3 ± 19.5		1.48 ± 0.42^a^		0.42 ± 0.14	
Obesity	51.2 ± 16.1		833 ± 130		31.6 ± 13.1		1.20 ± 0.31^b^		0.34 ± 0.96	
Skin type										
Very light or light	50.3 ± 13.0	0.698	756 ± 131	0.311	34.1 ± 18.8	0.470	1.38 ± 0.43	0.877	0.38 ± 0.13	0.946
Intermediate	52.6 ± 13.5		785 ± 152		28.9 ± 16.3		1.37 ± 0.37		0.39 ± 0.12	
Tanned	51.2 ± 14.5		814 ± 179		28.2 ± 11.2		1.31 ± 0.41		0.38 ± 0.13	
Brown or dark	48.5 ± 14.8		750 ± 155		28.3 ± 13.6		1.32 ± 0.43		0.37 ± 0.13	
Previous pregnancy										
0	50.1 ± 14.4	0.137	788 ± 112	0.136	27.4 ± 15.9	0.537	1.29 ± 0.36	0.136	0.37 ± 0.12	0.086
1	53.5 ± 14.2		774 ± 150		30.0 ± 14.3		1.43 ± 0.41		0.41 ± 0.13	
≥2	47.4 ± 12.5		772 ± 181		31.2 ± 14.8		1.27 ± 0.41		0.35 ± 0.13	
Gestational weight gain										
<0.30 kg/wk	50.2 ± 14.4	0.675	762 ± 133	0.238	28.6 ± 15.8	0.499	1.33 ± 0.41	0.792	0.38 ± 0.13	0.915
≥0.30 kg/wk	51.3 ± 13.6		795 ± 162		30.5 ± 13.9		1.35 ± 0.40		0.38 ± 0.12	
Season										
Spring	58.7 ± 13.1^a^	**<0.001**	841 ± 160^a^	**0.019**	31.1 ± 12.7	0.660	1.45 ± 0.38^a^	**0.014**	0.42 ± 0.12^a^	**0.002**
Summer	52.5 ± 13.4^ab^		761 ± 159^ab^		26.7 ± 15.0		1.42 ± 0.43^ab^		041 ± 0.13^ab^	
Autumn	48.4 ± 13.8^bc^		757 ± 120^ab^		29.1 ± 16.5		1.31 ± 0.41^ab^		0.36 ± 0.12^abc^	
Winter	41.1 ± 8.6^c^		735 ± 126^b^		30.6 ± 16.2		1.15 ± 0.33^b^		0.31 ± 0.99^c^	

^*∗*^Values are unadjusted mean ± standard deviation. *P*^†^Values for unadjusted least square means (LSD) post hoc test. For each exposure variable, different superscript letters indicate significant difference between categories on the outcome variable (25(OH)D_3_, DBP, PTH, free 25(OH)D_3_, and bioavailable 25(OH)D_3_).

**Table 3:** Linear regression data of variables associated with 25(OH)D_3_, DBP, PTH, free 25(OH)D_3_, and bioavailable 25(OH)D_3_ concentrations.

	25(OH)D_3_ (nmol/L)	DBP (mg/L)	PTH (pg/mL)	Free 25(OH)D_3_ (pg/mL)	Bioavailable 25(OH)D_3_ (ng/mL)
Independent variable	*β* (95% CI)	*β* (95% CI)	*β* (95% CI)	*β* (95% CI)	*β* (95% CI)
Gestational period, wk	**−0.73 (−1.31; −0.16)**	−5.91 (−12.54; 0.72)	0.03 (−0.67; 0.73)	−0.01 (−0.03; 0.00)	**−0.01 (−0.01; 0.00)**
Prepregnancy BMI, kg/m^2^	0.12 (−0.33; 0.57)	**5.92 (0.74; 11.09)**	0.10 (−0.44; 0.65)	−0.01 (−0.03; 0.00)	−0.00 (−0.01; 0.00)
Gestational weight gain, kg	0.14 (−0.32; 0.61)	0.87 (−4.47; 5.21)	0.25 (−0.31; 0.82)	0.01 (−0.01; 0.02)	0.00 (−0.00; 0.01)
Age, y	−0.20 (−0.66; 0.27)	−0.36 (−5.68; 4.95)	−0.70 (−0.63; 0.49)	−0.01 (−0.02; 0.01)	−0.00 (−0.01; 0.00)
ITA	0.05 (−0.08; 0.174)	−0.21 (−1.67; 1.25)	0.07 (−0.09; 0.22)	0.00 (−0.00; 0.01)	0.00 (−0.00; 0.00)
SEI hand	0.30 (−0.15; 0.74)	4.97 (−0.13; 10.07)	**−0.57 (−1.11; −0.30)**	−0.00 (−002; 0.01)	0.00 (−0.00; −0.01)
Number of previous pregnancies	−0.52 (−2.35; 1.30)	−6.63 (−27.66; 14.39)	1.23 (−0.99; 3.45)	0.01 (−0.05; 0.07)	−0.01 (−0.02; 0.01)
Season					
Spring	Ref	Ref	Ref	Ref	Ref
Summer	−4.78 (−11.21; 1.65)	−71.63 (−145.72; 2.47)	−5.11 (−12.94; 2.73)	0.00 (−0.20; 0.21)	0.00 (−0.07; 0.06)
Autumn	**−9.90 (−16.14; −3.64)**	**−86.78 (−158.77; −14.79)**	−0.80 (−8.41; 6.81)	−0.12 (−0.32; 0.08)	−0.05 (−0.11; 0.01)
Winter	**−16.74 (−23.13; −10.34)**	**−85.39 (−159.10; −11.69)**	−1.32 (−9.11; 6.48)	**−0.33 (−0.53; −0.13)**	**−0.11 (−0.17; −0.05)**

Results were obtained from multiple linear regression analysis with 25(OH)D_3_, DBP, PTH, free 25(OH)D_3_, and bioavailable 25(OH)D_3_ as dependent variables. ITA, individual typology angle; SEI, sun exposure index.

## Data Availability

The data used to support the findings of this study are available from the corresponding author upon request.

## Abbreviations

25(OH)D: 25-hydroxyvitamin D
1,25(OH)_2_D: 1,25-dihydroxyvitamin D
BMI: Body mass index
CI: Confidence interval
DAD: Diode array detector
DBP: Vitamin D-binding protein
HPLC-DAD: High-performance liquid chromatography coupled with diode array detector
iPTH: Intact parathyroid hormone
ITA: Individual typology angle
LSD: Least square means
*m*-DBP: Monoclonal antibody
*p*-DBP: Polyclonal antibody assay
PTH: Parathyroid hormone
SD: Standard deviation
SEI: Sun exposure index
UV: Ultraviolet.

## Supplementary Materials

Supplementary Materials Supplementary Table: concentrations of DBP, and free and bioavailable fractions of 25(OH)D_3_ estimated by polyclonal and monoclonal DBP assays.
