# Supplementary material for: Free and Bioavailable Fractions of Vitamin D: Association with Maternal Characteristics in Brazilian Pregnant Women
Source: J Nutr Metab. 2020 Sep 16;2020:1408659. doi: 10.1155/2020/1408659 (PMC7519195; doi:10.1155/2020/1408659)
Supplement: Supplementary Materials — Supplementary Table: concentrations of DBP, and free and bioavailable fractions of 25(OH)D3 estimated by polyclonal and monoclonal DBP assays. [file 1408659.f1.docx]

**Supplementary Table.** Concentrations of DBP and free and bioavailable fractions of 25(OH)D_3_ estimated by polyclonal and monoclonal DBP assays.

| Biochemical index | Polyclonal | Monoclonal | *P*-Value* |
| --- | --- | --- | --- |
|  | Mean ± SD | |  |
| DBP, mg/L | 773.5±129.5 | 347.3±63.3 | <0.001 |
| Free 25(OH)D_3_, pg/mL | 1.31±0.40 | 2.89±0.88 | <0.001 |
| Bioavailable 25(OH)D_3_, ng/mL | 0.37±0.13 | 0.82±0.30 | <0.001 |

**P*-value obtained by paired t test in a subsample of the pregnant women studied (n=52).
